# A matched cross-sectional study of the association between circulating tissue factor activity, immune activation and advanced liver fibrosis in hepatitis C infection

**DOI:** 10.1186/s12879-015-0920-1

**Published:** 2015-04-17

**Authors:** Aimee C Hodowanec, Rebecca D Lee, Kirsten E Brady, Weihua Gao, Stacey Kincaid, Jill Plants, Mieoak Bahk, Nigel Mackman, Alan L Landay, Gregory D Huhn

**Affiliations:** Ruth M. Rothstein CORE Center, 2020 W. Harrison St, Chicago, IL 60612 USA; Rush University Medical Center, Section of Infectious Diseases, Chicago, IL USA; University of North Carolina at Chapel Hill, Division of Hematology and Oncology, Chapel Hill, NC USA; Rush University Medical Center, Department of Immunology and Microbiology, Chicago, IL USA; Center for Clinical and Translational Sciences, University of Illinois at Chicago, Chicago, IL USA

**Keywords:** Viral hepatitis, Hepatitis C, HIV, Tissue factor, Fibrosis, Cirrhosis, Immune activation, Coagulation

## Abstract

**Background:**

Tissue factor (TF) is a protein that mediates the initiation of the coagulation cascade. TF expression is increased in patients with poorly-controlled HIV, and may be associated with increased immune activation that leads to cardiovascular morbidity. The role of TF in immune activation in liver disease in hepatitis C virus (HCV)-monoinfection and HIV/HCV-coinfection has not been explored.

**Methods:**

Fifty-nine patients were stratified: A) HIV-monoinfection (N = 15), B) HCV-monoinfection with chronic hepatitis C (CHC) (N = 15), C) HIV/HCV-coinfection with CHC (N = 14), and D) HIV/HCV-seropositive with cleared-HCV (N = 15). All HIV+ patients had undetectable HIV viremia. Whole blood was collected for CD4/CD8 immune activation markers by flow cytometry and plasma was assayed for microparticle TF (MPTF) activity. Subjects underwent transient elastography (TE) to stage liver fibrosis*.* Undetectable versus detectable MPTF was compared across strata using Fisher's Exact test.

**Results:**

MPTF activity was more frequently detected among patients with HCV-monoinfection (40%), compared to HIV-monoinfection and HIV/HCV-seropositive with cleared HCV (7%) and HIV/HCV-coinfection with CHC (14%) (p = 0.02). Mean TE-derived liver stiffness score in kPa was higher in patients with detectable MPTF (12.4 ± 8.5) than those with undetectable MPTF (6.4 ± 3.0) (p = 0.01). Mean CD4 + HLADR+ and CD4 + CD38-HLADR+ expression were higher in those with detectable MPTF (44 ± 9.8% and 38 ± 8.7%, respectively) than those with undetectable MPTF (36 ± 11% and 31 ± 10.4% respectively) (p = 0.05 and 0.04 respectively).

**Conclusions:**

HCV-monoinfection and HIV/HCV-coinfection with CHC were associated with MPTF activity. MPTF activity is also associated with advanced liver fibrosis and with CD4 + HLADR+ immune activation.

## Background

Hepatitis C virus (HCV) is a leading cause of morbidity and mortality among HIV-infected individuals [1]. Persons coinfected with HIV and HCV are at risk for more rapid progression of liver disease as compared to those with HCV-monoinfection [2,3]. The pathogenesis of the accelerated liver fibrosis observed in HIV/HCV-coinfected patients is complex and incompletely understood. The cellular immune system is vital in the control of HCV infection and it has been postulated that the HCV-specific immune response in HIV-infected persons is impaired and deregulated. This notion is supported by the finding that HIV/HCV-coinfected patients with low CD4+ T cell counts are more likely to experience accelerated fibrosis [4,5]. Data regarding the pathogenesis of liver fibrosis among co-infected patients with well-controlled HIV are lacking.

HIV-associated immune activation is known to play an integral role in HIV pathogenesis and likely contributes to the pathogenesis of liver fibrosis in HIV/HCV-coinfected patients as well. Chronic immune activation results in a pro-coagulant state. One mechanism of increased coaguability in the setting of immune activation involves increased tissue factor (TF) expression in response to tissue damage and cytokine release [6]. TF is a transmembrane protein that mediates thrombin signaling in the activation of the coagulation cascade. TF exists in several states: as cell-free TF in plasma, on cell surfaces, and on cell-derived microparticles (MPs) [7]. MPs are membrane vesicles that are released from activated or apoptotic cells. Activation of cells or platelets by systemic inflammation initiates an innate immune pathway whereby plasma membrane blebs are released and enter into the circulation, exposing procoagulant phosphatidylserine and cellular epitopes conferring functionality [8]. MPs are particularly prothrombotic when they express TF [9,10]. Although it represents a small portion of circulating TF, microparticle TF (MPTF) is believed to be a functionally active form of TF. TF has been implicated in a variety of disease states, including malignancy, cardiovascular disease, acute liver injury, septic shock, and HIV [11-13]. In HIV infected patients, increased TF expression has been found to be associated with increased HIV viral load, hepatitis B or C coinfection, markers of lipopolysaccharide (LPS) exposure, and importantly, markers of immune activation [7,14]. The impact of TF on immune activation and liver fibrosis among HCV infected patients has not been extensively explored. In this study, we examine the relationship between circulating MPTF activity and immune activation markers and their association with the development of advanced liver fibrosis among HCV monoinfected patients and patients co-infected with well-controlled HIV. Patients with suppressed HIV viremia are the focus of this study because data on TF, immune activation, and liver fibrosis are limited in this contemporary HIV population.

## Methods

### Study design and population

This cross-sectional study was conducted at the Ruth M. Rothstein CORE Center in Chicago, IL between June 2011 and December 2011 and was approved by The Cook County Health and Hospitals System Institutional Review Board. Written, informed consent was obtained from each subject. Participants were stratified according to HIV and HCV status, as follows: A) HIV-monoinfection (N = 15), B) HCV-monoinfection with CHC (N = 15), C) HIV/HCV-coinfection with chronic hepatitis C (CHC) (N = 14), and D) HIV/HCV-seropositive with cleared-HCV (N = 15). Strata D consisted of both patients with spontaneous HCV clearance (N = 11) and patients who achieved a sustained virologic response following HCV therapy (N = 4). As a pilot study, sample size was determined by feasibility. Strata D was enrolled first to establish matching criteria. Subjects were matched by age, sex, estimated duration of HIV and HCV infection (HIV duration was estimated based upon self-reported year of probable high-risk exposure and if that exposure time point was unknown, then the date of diagnosis was used; HCV duration was calculated based on self-reported year of onset of high-risk activity (tattoo, exchange of sex for drugs, etc.) and if there was no clear exposure, the duration of HCV was reported as unknown), and CD4 count, with current CD4 counts in strata A and C being matched to CD4 at estimated time point of HCV clearance in strata D. All HIV-positive patients had undetectable HIV RNA. Each subject completed a questionnaire regarding HIV and HCV risk factors, had blood collected, and underwent assessment of liver fibrosis. Through the questionnaire and chart abstraction, data was collected regarding demographic factors (ethnicity), lifestyle factors (history of IDU, alcohol use, coffee consumption, smoking), and labs (D-dimer, high-sensitivity c-reactive protein (hsCRP), quantitative cytomegalovirus immunoglobulin G, complete blood count, complete metabolic panel, coagulation tests, and lipid profile) to allow for identification of potential covariates.

Liver stiffness was assessed using transient elastogroaphy (TE), a non-invasive means of assessing liver fibrosis by transmitting low speed elastic waves (~1 m/sec) and determining their propagation velocity with high speed ultrasound. All TE operators were trained and certified. Measurements were obtained via an ultrasonic probe (placed in the intercostal space at the level of the xiphoid process) perpendicular to the skin overlying the right lobe of the liver. The TE-measured liver stiffness was expressed in kilopascals (kPa) and determined from the median value of 10 “valid” measurements (ie, elastic waves that are propagated through the liver according to the machine software). The speed of wave propagation through approximately 1/500^th^ the volume of the liver is directly correlated with the square root of the elastic modulus or tissue stiffness [15,16]. TE-derived fibrosis scores correlate with Metavir fibrosis staging system as follows: <7.1 = stage 0–1, 7.1-9.4 = stage 2, 9.5-12.4 = stage 3, and ≥ 12.5 = stage 4. The fibrosis stage predicted by TE has been shown to correlate with the histologically determined fibrosis stage [17]. We reported liver stiffness by strata and examined its relationship with TF and immune activation markers by comparing no-moderate fibrosis (stage 0–2) to severe fibrosis (stage 3–4).

### Laboratory methods

Peripheral blood was collected into heparin, EDTA, sodium citrate, and serum-separator tubes (SST) via venipuncture from patients. EDTA and sodium citrate tubes were processed within one hour. After centrifugation serum was collected from the SST tube and frozen at −80°C. Peripheral blood mononuclear cells (PBMC) were isolated from heparin tubes by density gradient centrifugation using lymphocyte separation medium (Mediatech, Inc). Cells were cryopreserved in freeze media (90% FBS + 10% DMSO) and stored in liquid nitrogen (LN_2_) until testing.

Characterization of T cell immune activation and memory was performed in batch by polychromatic flow cytometry on frozen/thawed heparin PBMCs. Frozen PBMCs were removed from LN_2_ storage and thawed rapidly in a 37°C water bath, washed, and rested overnight in a 37°C incubator. The following day cells were washed and stained for cell viability with Aqua Live/Dead cell stain kit (Invitrogen) prior to cell surface staining. Cell surface markers were stained with fluorochrome-conjugated monoclonal antibodies to CD3, CD8, HLA-DR, CD38, CD45RA, CCR7 (BD Biosciences), and CD4 (Invitrogen). After staining, cells were fixed in 2% formaldehyde and analyzed within 24 hours on a LSR2 flow cytometer (BD) using FACS Diva software v6.1.1. Analysis of flow cytometry data was performed using FlowJo software (Tree Star Inc). Immune activation (CD38^+^\HLA DR^+^) and memory (CD45RA\CCR7) analyses were performed after stringent gating on singlet live (Aqua^−^) CD3^+^\CD4^+^ or CD3^+^\CD8^+^ T cells.

EDTA plasma specimens were assayed in duplicate for circulating levels of IFNγ, IL-1β, IL-6, IL-8, IL-10, IL-12(p40), IL-12(p70), IL-15, IL-17a, IP-10, MIP-1α, and TNF-α using the Human Cytokine/Chemokine kit (EMD Millipore, Billerica, MA) according to manufacturer recommended protocols. All kits were read using a Luminex 100 IS System (Luminex Corp, Austin, TX) by the Rush Proteomics and Biomarkers Core Facility with biomarker concentrations calculated using a 5-parametric curve fit using xPonent 3.2 software (Luminex Corp). Median %CV and assay recovery values all fell within acceptable limits specified by EMD-Millipore.

### Plasma Preparation and MPTF Activity Measurement

Plasma specimens were collected using sodium citrate tubes and spun at 1500 × g for 15 minutes at room temperature. Platelet-poor plasma (PPP) was removed from the vacutainer tube and placed in a 15 mL conical tube and spun down a second time at 1500 × g for 15 minutes to obtain platelet-free plasma (PFP), which was transferred to a new 15 mL conical tube and vortexed to thoroughly mix. The plasma was aliquoted into 0.5 mL cryovials, flash frozen in LN_2_, and stored at −80°C until analyzed.

MPTF activity was measured by a two-stage chromogenic assay as previously reported [18]. MPs were pelleted from 200 μL of PFP by centrifugation at 20 000 × *g* for 15 min at 4°C, washed twice with HBSA (137 mm NaCl, 5.38 mm KCl, 5.55 mm glucose, 10 mm HEPES, 0.1% bovine serum albumin, pH 7.5), and re-suspended in 100 μL of HBSA. Samples were incubated with either 1 μL of a TF-blocking antibody (hTF1) (4 μg/mL) or 1 μL of a control antibody (mouse IgG: 4 μg/mL) for 15 minutes at 25°C and then 50 μL aliquots were added to duplicate wells of a 96-well plate. Each MP sample was then incubated for 2 hours at 37°C in a 50 μL mixture of HBSA containing 10 nm factor VIIa, 300 nm factor X and 10 mm CaCl_2_. Factor Xa generation was stopped by the addition of 25 μL of 25 mm EDTA buffer and 25 μL of the chromogenic substrate S2765 (4 mm) was added and incubated at 37°C for 15 min. Finally, absorbance at 405 nm was measured using a VERSAmax microplate reader (Molecular Devices). TF activity was calculated by reference to a standard curve generated using relipidated recombinant human TF (0–55 pg/mL). The TF-dependent factor Xa generation (pg/mL) was determined by subtracting the amount of factor Xa generated in the presence of hTF1 from the amount of factor Xa generated in the presence of the control antibody.

### Statistical analyses

The number of patients with detectable MPTF activity versus no detectable MPTF activity (undetectable) were compared among subjects with HCV-monoinfection (strata B), those with HIV/HCV-coinfection with CHC (strata C) and those with resolved or no hepatitis C (strata A and D) using Fisher's Exact test. In addition, the quantitative detectable TF levels were compared between the strata using ANOVA. For normally distributed data, two sample t test was used to compare the difference between undetectable and detectable TF; for non-normally distributed data, Wilcoxon Rank-Sum test was used to compare the difference between undetectable and detectable TF. Mean and standard deviation for normal data, median (minimum, maximum) for non-normal data, number and percentage for categorical data were reported. P-values <0.05 were considered statistically significant. All analyses were performed using SAS Version 9.2 (Cary, NC).

## Results

A total of 63 subjects were enrolled; 4 patients who did not meet inclusion criteria were subsequently excluded (3 were HIV-positive patients who were found to have positive HIV RNA and 1 was an HIV/HCV coinfected patient initially enrolled for strata C but found to have negative HCV RNA) and the remaining 59 subjects were analyzed (Table 1). Among HIV-positive subjects, the median CD4 count was 420 (295–1117) cells/μl. The estimated median duration of HCV infection among strata B, C, and D was 26 (3–40) years.

**Table 1:** **Baseline characteristics of HIV-Infected, HCV-Infected, and HIV/HCV-coinfected patients (n = 59)**

**Variable**	**Strata A (N = 15)**	**Strata B (N = 15)**	**Strata C (N = 14)**	**Strata D (N = 15)**	***P***
**Demographics**					
**Sex, N (%)**					1.00
**Female**	4 (27)	4 (27)	4 (29)	4 (27)	
**Male**	11 (73)	11 (73)	10 (71)	11 (73)	
**Age, years**	53 (51–56)	54 (51–56)	54 (52–56)	53 (42–66)	0.95
**Race/Ethnicity, N (%)**					0.21
**African American/non-Hispanic**	11 (73)	12 (80)	9 (64)	13 (87)	
**Caucasian/non-Hispanic**	4 (27)	2 (13)	4 (29)	0	
**Caucasian/Hispanic**	0	1 (7)	1 (7)	2 (13)	
**Laboratory values**					
**ALT (U/L)**	18(11–82)	43 (19–78)	23(14–88)	22 (10–77)	0.0009
**AST (U/L)**	19 (14–52)	37 (23–84)	30 (18–83)	24 (18–55)	0.0002
**Albumin (g/dL)**	4.2 (3.8-5.1)	4.0 (3.4-4.7)	4.3 (2.7-4.7)	4.2 (3.8-5.0)	0.29
**Total cholesterol (mg/dL)**	168 (118–253)	155 (88–200)	147.5 (98–207)	171 (124–220)	0.24
**hs-CRP (mg/L)**	4.0 (0.5-11.3)	1.2 (0.2-36.9)	0.7 (0.2-27.2)	3.5 (0.2-65.3)	0.08
**D-dimer (μg/mL)**	0.34 (0.19-0.98)	0.34 (0.21-2.25)	0.22 (0.19-2.07)	0.35 (0.19-1.49)	0.41
**Risk factors, N (%)**					
**Sexual preference**					0.01
**MSM**	6 (40)	1 (7)	1 (7)	1 (7)	
**Heterosexual**	7 (47)	14 (93)	11 (79)	14 (93)	
**Bisexual**	2 (13)	0	2 (14)	0	
**Alcohol use, N (%)**					0.32
**> once a week**	4 (27)	1 (7)	2 (14)	0	
**< weekly, but > monthly**	2 (13)	1 (7)	2 (14)	1 (7)	
**Monthly or less**	9 (60)	13 (86)	10 (72)	14 (93)	
**Injection drug use ever, N (%)**					<0.0001
**Yes**	1 (7)	9 (60)	13 (93)	9 (60)	
**No**	14 (93)	6 (40)	1 (7)	6 (40)	
TE-derived fibrosis score*	4.4 (3.0-11.6)	7.9 (4.7-29.9)	7.6 (3.2-22.3)	5.9 (3.0-9.1)	0.001
**Current CD4 count (cells/μL)**	495 (296–956)	-	457 (299–651)	668 (295–1117)	0.01
**Nadir CD4 count (cells/μL)**	168 (22–415)	-	225 (13–428)	302 (124–918)	0.08
**Duration of ARVs (years)**	9 (1–25)	-	11(3–15)	6 (0–20)	0.59
**Estimated duration of HIV (years)**	12 (2–25)	-	17 (5–31)	19 (1–28)	0.69
**Estimated duration of HCV (years)**	-	24 (15–40)	30 (12–38)	26 (3–37)	0.23
**HCV viral load (IU/mL)**	-	974,248 (1,622-4,604,960)	706,476 (17,145-20,000,000)	-	0.95

Values are expressed as median (range) unless otherwise noted. *TE-derived fibrosis scores correlate with Metavir fibrosis staging system as follows: <7.1 = stage 0–1, 7.1-9.4 = stage 2, 9.5-12.4 = stage 3, and ≥ 12.5 = stage 4.

Strata: A = HIV monoinfection, B = HCV monoinfection, C = HIV/HCV coinfection with CHC, D = HIV/HCV coinfection with cleared HCV.

Abbreviations: ARV, antiretroviral; hsCRP, high sensitivity C-reactive protein; MSM, men who have sex with men; TE, transient elastography.

Patients with HCV-monoinfection were more likely to have detectable MPTF (40%), compared to HIV-monoinfection and HIV/HCV-seropositive with cleared HCV (7%) and HIV/HCV-coinfection with CHC (14%) (p = 0.02) (Table 2). Among subjects with detectable MPTF, there was no statistically significant difference in the mean MPTF activity (pg/ml) between strata (A and D = 0.31 ± 0.32, B = 0.15 ± 0.21, C = 0.14 ± 0.05; p = 0.42). Further, MPTF activity was found to be associated with liver fibrosis. The mean TE-derived liver stiffness score in kPa was higher in patients with detectable MPTF (12.4 ± 8.5) than those with undetectable MPTF (6.4 ± 3.0)(p = 0.01). Subjects with TE-derived stage F3-4 on METAVIR scale were more likely to have detectable MPTF than those with F0-2 (p = 0.03) (Table 2).

**Table 2:** **Tissue factor detection by strata and fibrosis stage**

	**TF Undetectable**	**TF Detectable**	**Total**	**p-value**
**Strata**				0.02
**A and D**	28 (93%)	2 (7%)	30	
**B**	9 (60%)	6 (40%)	15	
**C**	12 (86%)	2 (14%)	14	
**Fibrosis stage**				0.03
**0 - 2**	40 (89%)	5 (11%)	45	
**3 - 4**	7 (58%)	5 (42%)	12	
**Mean ± SD TE-derived liver stiffness (kPa)**	6.4 ± 3.0	12.4 ± 8.5		0.01

Strata A: HIV-monoinfection, Strata B: HCV-monoinfection, Strata C: HIV/HCV-coinfection with CHC, and Strata D: HIV/HCV-seropositive with cleared HCV.

*Abbreviations:* *CHC* chronic hepatitis C, *TE* transient elastography, *TF* tissue factor.

Across strata, the mean percentage of CD4+ T cells expressing the immune activation markers HLADR+ and CD38-HLADR+ was higher in those with detectable MPTF (44 ± 9.8% and 38 ± 8.7% respectively) than those with undetectable MPTF (36 ± 11% and 31 ± 10.4% respectively)(p = 0.05 and 0.04 respectively). The mean percentage of CD4 T cells of the terminal effectors expressing RA subtype was lower in patients with detectable MPTF (8 ± 6.3%) compared to those with undetectable MPTF (13 ± 6.8%, p = 0.04) (Table 3). There was no statistically significant difference in the expression CD8+ T cell markers of immune activation, memory subsets, or cytokine levels among those with and without detectable MPTF. Among HCV-monoinfected subjects, there were no significant differences in immune activation or inflammation markers stratified by detectable versus undetectable MPTF, though there was a trend towards lower mean IL-10 levels in subjects with detectable compared to undetectable MPTF (1.69 ± 2.84 vs. 2.86 ± 2.29 pg/mL; p = 0.07).

**Table 3:** **Association between tissue factor detection and CD4 T-cell immune activation**

**CD4 T-cell marker**	**Percent expression in patients with:**		***p*** **-value**
	**Detectable TF**	**Undetectable TF**	
HLADR+	43.8 ± 9.8	36.2 ± 11.0	0.05
TotalCD38+	15.2 ± 6.4	19.5 ± 9.1	0.16
CD38 + HLADR+	5.7 ± 2.9	5.5 ± 2.8	0.85
CD38-HLADR+	38.2 ± 8.7	30.7 ± 10.4	0.04
CD38 + HLADR-	9.6 ± 3.7	14.0 ± 7.4	0.01
CD45RA + CCR7-	8.3 ± 6.3	13.3 ± 6.8	0.04
CD45RA-CCR7+	19.9 ± 5.2	19.9 ± 5.2	0.99
CD45RA + CCR7+	18.4 ± 12.1	18.8 ± 10.1	0.91
CD45RA-CCR7-	53.4 ± 15.4	48.0 ± 12.3	0.23

Results reported as mean ± standard deviation.

CD45RA + CCR7-, terminal effectors expressing RA (TEMRA); CD45RA-CCR7+, Central Memory; CD45RA + CCR7+, naïve; CD45RA-CCR7-, effector memory; TF, tissue factor

Lastly, there was no difference in hsCRP level in those with detectable MPTF activity as compared to those without detectable MPTF activity (median: 3.9 (0.2 - 65.3) mg/L vs.1.9 (0.2 - 36.9) mg/L, respectively, p = 0.41). Similarly, the D-dimer did not differ significantly between those with detectable MPTF activity and those without detectable MPTF activity (median: 0.35 (0.2 – 1.0) μg/mL vs. 0.32 (0.2 – 2.3) μg/mL, respectively, p = 0.65).

## Discussion

In this exploratory study, we found that the presence of MPTF is associated with advanced fibrosis in our cohort of patients with HIV, HCV, and HIV/HCV coinfection. Accordingly, the strata including patients with active HCV were more likely to have detectable circulating MPTF than patients in the strata without active HCV. Circulating MPTF activity was also found to be associated with CD4 HLADR+ T-cell immune activation. Data previously reported from our cohort demonstrated significantly increased CD4+ HLADR+ levels in HIV/HCV coinfection with CHC compared to HIV/HCV coinfection with cleared HCV and HIV monoinfection, though not significantly different from HCV monoinfection, suggesting that CD4 + HLADR+ expression is driven by HCV viremia [19]. MPTF detection was greatest among HCV monoinfected patients compared to HIV/HCV coinfected patients, a finding which may reflect a down-regulation of TF-associated immune responses due to antiviral control of HIV in HIV/HCV coinfection that is absent patients with HCV monoinfection [20]. We also demonstrated higher levels of effector memory CD4 cells expressing CD45RA+ and CD4+ CD38 + HLADR- expression in subjects with undetectable TF compared to detectable TF, however at lower magnitudes overall than other effector, memory, naïve, and immune activation CD4 cell subsets. In HIV-infected patients with virologic suppression, the representative HIV-infected population in our cohort, a recent report identified no differences in CD4+ naïve, central memory, and effector cell subsets in patients with protease-activated receptor 1 expression, a thrombin activated receptor that functions as a proinflammatory chemokine regulator, compared to healthy HIV-negative controls, further suggesting that potential TF-mediated immune activation is attenuated in the setting of well-controlled HIV [21].

Systemic inflammation mediated by monocyte and T cell immune activation may be an important link between MPTF activity and liver fibrosis. Funderburg et al. demonstrated that expression of TF on monocytes was associated with immune activation and with expression of soluble CD14, the LPS receptor released following translocation with LPS stimulation in HIV infection [14]. In addition, LPS, a stimulus known to induce MP release from monocytes and macrophages, has been shown to be associated with liver fibrosis in patients with HCV-related liver disease, though the mechanism by which LPS contributes to liver fibrosis is not well understood [22-24]. In advanced fibrosis, the increase in proinflammatory cytokines in the plasma could induce the release of MPs by endothelial cells. MPs derived from activated and apoptotic T cells can fuse with cell membranes of hepatic stellate cells (HSCs), the major effector cells for extracellular matrix deposition in liver fibrosis, and induce fibrolytic activation in a negative feedback loop in HSCs [25]. In chronic hepatitis C, inhibition of apoptosis of HSCs is associated with advanced fibrosis [26-28]. Within our cohort, we have previously observed a trend toward lower stage fibrosis with higher IL-10 levels, a pleiotropic anti-inflammatory cytokine (19). Mouse models have shown that IL-10 inhibits LPS induction of TF in macrophages [29]. Similarly, in our current analysis, we found that among hepatitis C monoinfected subjects, those with detectable TF had a trend toward lower IL-10 levels. Given the possible association between TF production and liver fibrosis, this finding further supports a potential inverse relationship between IL-10 and liver fibrosis. As apoptotic cells are one of the sources of circulating MPs, the impact of functional TF expression on the complex immunopathogenesis of liver fibrosis is likely a balance between endothelial cell apoptosis and inflammatory monocyte/macrophage origins of MPTF in viral infection [30]. Our current cohort’s findings suggest that TF as an innate immune activation component may play a role in the development of advanced liver fibrosis in CHC.

As TF may represent a biomarker for liver fibrosis in patients with CHC, the TF and thrombin pathways may serve as therapeutic targets aimed at preventing or slowing liver fibrosis. Murine models have been developed to demonstrate that targeted inhibition of TF and thrombin can reduce liver fibrosis [31-33]. Whether the safe use of thrombin inhibitors may confer TF-associated anti-fibrotic activity in patients with hepatitis C-related liver fibrosis requires validation with measures of TF activity in larger interventional studies. Additionally, epidemiological studies centered on traditional metabolic and behavioral risk factors, as well as carotid artery assessments, have established a potential link between HCV and increased CVD risk [34-37]. HCV RNA has also been detected in carotid plaques in asymptomatic patients with chronic HCV [38]. Investigations of emerging anti-fibrotic agents, such as monoclonal antibodies against regulators of fibrogenesis, Toll-like receptors, and caspase inhibitors, which incorporate endothelial biomarker analyses such as TF activity may generate important translational information, with implications for possible therapeutic approaches, that may further correlate the intersection of inflammatory responses to hepatitis C infection, liver fibrosis, and CVD risk [39].

There were several limitations of this study. First, our sample size was small; We attempted to account for important factors in the natural history of hepatic fibrogenesis by enrolling subjects that were well-matched by age, gender, CD4 count, and estimated duration of HIV and HCV infection. However, the small cohort precluded the performance of a multivariate analysis which may have limited our ability to control for other relevant variables. Second, MPTF levels were below the limit of detection in the majority of study subjects’ plasma specimens. This restricted our ability to perform meaningful analysis based upon differences in levels of MPTF by HIV and HCV infection status. However, this finding was not unexpected as undetectable MPTF measurements are common in adults without acute inflammatory or active cardiovascular comorbidities [40]. In addition, our use of PFP to measure MPTF activity may have contributed to lower levels of MPTF compared to levels reported in recent studies of TF using PPP from HIV-infected and CHC patients [7,11,25]. However, we felt this was necessary to preclude possible exposure to platelets, which may result in increased MPTF activity because of TF reactogenicity to anionic phospholipids on the surface of platelets [41-43]. We also recognize that our methodology for assaying MPTF activity cannot determine the cellular phenotype of TF, though the vast majority of TF in blood is thought to originate from activated monocytes [44,45]. Thus, we are not able to determine if innate monocyte activation is driving TF activity in our subjects. Similarly, the cross-sectional study design cannot specifically address whether elevated MPTF expression in HCV are mediators of a disease process contributing to liver fibrosis or increased cardiovascular risk or simply a consequence of enhanced global inflammation associated a persistent viral infection without direct causality. Alternatively, the observed association between MPTF and liver fibrosis may reflect impaired MP clearance in the setting of cirrhosis because the liver, with other organs, contributes to MP clearance [46-50]. Longitudinal studies determining whether interventions along the TF-induced coagulation pathway may exert anti-fibrotic activity and impact CVD endpoints are needed.

## Conclusions

The pathogenesis of HCV-associated liver fibrosis is complex and multifaceted. Coinfection with HIV may accelerate this process, but the dynamic immunologic mechanisms by which this occurs remain incompletely understood. Findings of our study contribute further data suggesting that immune activation plays a role in the development of liver fibrosis. In addition, we identify TF as a possible biomarker for advanced liver fibrosis in chronic hepatitis C infection. The potential diagnostic and therapeutic applications of TF among patients with liver disease warrant further studies.

## Abbreviations

HCV: Hepatitis C virus
TF: Tissue factor
MP: Microparticle
MPTF: Microparticle tissue factor
LPS: Lipopolysaccharide
CHC: Chronic hepatitis C
CHC: Chronic hepatitis C
IDU: Injection drug use
hsCRP: High sensitivity C reactive protein
TE: Transient elastography
SST: Serum separator tube
PBMC: Peripheral blood mononuclear cells
LN_2_: Liquid nitrogen
PPP: Platelet-poor plasma
PFP: Platelet-free plasma

## References

[1] Weber R, Sabin CA, Friis-Møller N, Reiss P, El-Sar WM, Kirk O (2006). Liver Related Deaths in Persons Infected with the HIV Virus: The D-A-D Study. Ann Intern Med.

[2] Graham CS, Baden LR, Yu E, Mrus JM, Carnie J, Heeren T (2001). Influence of human immunodeficiency virus infection on the course of hepatitis C virus infection: a meta-analysis. Clin Infect Dis.

[3] Soto B, Sanchez-Quijano A, Rodrigo L, Del Olmo JA, García-Bengoechea M, Hernández-Quero J (1997). Human immunodeficiency virus infection modifies the natural history of chronic parenterally-acquired hepatitis C with an unusually rapid progression to cirrhosis. J Hepatol.

[4] Di Martino V, Rufat P, Boyer N, Renard P, Degos F, Martinot-Peignoux M (2001). The influence of human immunodeficiency virus coinfection on chronic hepatitis C in injection drug users: a long-term retrospective cohort study. Hepatology.

[5] Monto A, Kakar S, Dove LM, Bostrom A, Miller EL, Wright TL (2006). Contributions to hepatic fibrosis in HIV-HCV coinfected and HCV monoinfected patients. Am J Gastroenterol.

[6] Baker JV. Chronic HIV disease and activation of the coagulation system. Thromb Res. 2013;132(5):495–9. 10.1016/j.thromres.2013.08.016 PMC4197841 24034985

[7] Baker JV, Hullsiek KH, Bradford RL, Prosser R, Tracy RP, Key NS (2013). Circulating levels of tissue factor microparticle procoagulant activity are reduced with antiretroviral therapy and are associated with persistent inflammation and coagulation activation among HIV positive patients. J Acquir Immune Defic Syndr.

[8] Coughlin SR (2000). Thrombin signaling and protease-activated receptors. Nature.

[9] Key NS (2010). Analysis of tissue factor positive microparticles. Thromb Res.

[10] Morel O, Jesel L, Freyssinet JM, Toti F (2011). Cellular mechanisms underlying the formation of circulating microparticles. Arterioscler Thromb Vasc Biol.

[11] Chu AJ. Tissue factor, blood coagulation, and beyond: An overview. International J Inflammation 2011:367284. Epub. 10.4061/2011/367284 PMC3176495 21941675

[12] Stravitz RT, Bowling R, Bradford RL, Key NS, Glover S, Thacker LR (2013). Role of procoagulant microparticles in mediating complications and coutcome of acute liver injury/acute liver failure. Hepatology.

[13] Meziani F, Delabranche X, Asfar P, Toti F (2010). Bench-to-bedside review: circulating microparticles—a new player in sepsis?. Crit Care.

[14] Funderburg NT, Mayne E, Sieg SF, Asaad R, Jiang W, Kalinowska M (2010). Increased tissue factor expression on circulating monocytes in chronic HIV infection: relationship to in vivo coagulation and immune activation. Blood.

[15] Sandrin L, Tanter M, Catheline S, Fink M (2002). Shear modulus imaging with 2-D transient elastography. IEEE Trans Ultrason Ferroelectr Freq Control.

[16] Sandrin L, Tanter M, Gennisson JL, Catheline S, Fink M (2002). Shear elasticity probe for soft tissues with 1-D transient elastography. IEEE Trans Ultrason Ferroelectr Freq Control.

[17] Kirk GD, Astemborski J, Mehta SH, Spoler C, Fisher C, Allen D (2009). Assessment of liver fibrosis by transient elastography in persons with hepatitis C virus infection or HIV-hepatitis C virus coinfection. Clin Infect Dis.

[18] Khorana AA, Francis CW, Menzies KE, Wang JG, Hyrien O, Hathcock J (2008). Plasma tissue factor may be predictive of venous thromboembolism in pancreatic cancer. J Thromb Haemost.

[19] Hodowanec A, Brady K, Gao W, Kincaid S, Plants J, Bahk M (2013). Differences in CD4+ T-Cell Immune Activation in HIV, HCV, and HIV/HCV Coinfection are Influenced by HIV and HCV Infection Status. J Aquir Immune Defic Syndr.

[20] Catalfamo M, Wilhelm C, Tcheung L, Proschan M, Friesen T, Park JH (2011). CD4 and CD8 T Cell Immune Activation during Chronic HIV Infection; roles of Homeostasis, HIV, Type I IFN, IL-7. J Immunol.

[21] Hurley AH, Smith M, Karpova T, Hasley RB, Belkina N, Shaw S (2013). Enhanced Effector Function of CD8+ T Cells From Healthy Controls and HIV-infected Patients Occurs through Thrombin Activation of Protease-Activated Receptor 1. J Infect Dis.

[22] Balagopal A, Philp FH, Astemborski J, Block TM, Mehta A, Long R (2008). Human immunodeficiency virus-related microbial translocation and progression of hepatitis C. Gastroenterology.

[23] Rasaratnam B, Kaye D, Jennings G, Dudley F, Chin-Dusting J (2003). The effect of selective intestinal decontamination on the hyperdynamic circulatory state in cirrhosis. A randomized trial. Ann Intern Med.

[24] Gauley J, Pisetsky DS (2010). The release of microparticles by RAW 264.7 macrophage cells stimulated with TLR ligands. J Leukoc Biol.

[25] Kornek M, Popov Y, Libermann TA, Afdhal NH, Schuppan D (2011). Human T cell microparticles circulate in blood of hepatitis patients and induce fibrolytic activation of hepatic stellate cells. Hepatology.

[26] Gonzalez SA, Fiel MI, Sauk J, Canchis PW, Liu RC, Chiriboga L (2009). Inverse association between hepatic stellate cell apoptosis and fibrosis in chronic hepatitis C virus infection. J Viral Hepat.

[27] Friedman SL (2000). Molecular regulation of hepatic fibrosis, and integrated cellular response to tissue injury. J Biol Chem.

[28] Canbay A, Friedman S, Gores GJ (2004). Apoptosis: the nexus of liver injury and fibrosis. Hepatology.

[29] Baker AK, Wang R, Mackman N, Luyendyk JP (2009). Rapamycin enhances LPS induction of tissue factor and tumor necrosis factor-alpha expression in macrophages by reducing IL-10 expression. Mol Immunol.

[30] Antoniak S, Owens AP, Baunacke M, Williams JC, Lee RD, Weithäuser A (2013). PAR-1 contributes to the innate immune response during viral infection. J Clin Invest.

[31] Petts G, Dhar A, Kudo H, Sadiq F, Anstee QM, Kallis YN, et al. Targeted inhibition of tissue factor suppresses hepatic fibrosis in CCL4 treated mice. Gut. 2012;61:A28–8.

[32] Martinelli A, Knapp S, Anstee Q, Worku M, Tommasi A, Zucoloto S (2008). Effect of a thrombin receptor (protease-activated receptor 1, PAR-1) gene polymorphism in chronic hepatitis C liver fibrosis. J Gastroenterol Hepatol.

[33] Anstee QM, Goldin RD, Wright M, Martinelli A, Cox R, Thursz MR (2008). Coagulation status modulates murine hepatic fibrogenesis: implications for the development of novel therapies. J Thromb Haemost.

[34] Butt AA, Xiaoqiang W, Budoff M, Leaf D, Kuller LH, Justice AC (2009). Hepatitis C Virus Infection and the Risk of Coronary Disease. Clin Infect Dis.

[35] Petta S, Torres D, Fazio G, Cammà C, Cabibi D, Di Marco V (2012). Carotid Atherosclerosis and Chronic Hepatitis C: A Prospective Study of Risk Associations. Hepatology.

[36] Vassalle, Masini S, Bianchi F, Zucchelli GC (2004). Evidence for association between hepatitis C virus seropositivity and coronary artery disease. Heart.

[37] Alyan O, Kacmaz F, Ozdemir O, Deveci B, Astan R, Celebi AS (2008). Hepatitis C Infection is associated with increased coronary artery atherosclerosis defined by Modified Reardon Severity Score System. Circ J.

[38] Boddi M, Abbate R, Chellini B, Giusti B, Giannini C, Pratesi G (2010). Hepatitis C virus RNA localization in human carotid plaques. J Clin Virol.

[39] Talal AH, Feron-Rigodon M, Madere J, Subramanian GM. Simtuzumab, an Antifibrotic Monoclonal Antibody Against Lysyl Oxidase-Like 2 (LOXL2) Enzyme, Is Safe and Well Tolerated in Patients with Liver Disease. 2013 European Association of the Study of the Liver Conference. Abstract 1319.

[40] Owens AP, Mackman N (2011). Microparticles in hemostasis and thrombosis. Circ Res.

[41] Lee RD, Barcel DA, Williams JC, Wang JG, Boles JC, Manly DA (2012). Pre-analytical and analytical variables affecting the measurement of plasma-derived microparticle tissue factor activity. Thromb Res.

[42] Auwerda JJ, Yuana Y, Osanto S, de Maat MP, Sonneveld P, Bertina RM (2011). Microparticle-associated tissue factor activity and venous thrombosis in multiple myeloma. Thromb Haemost.

[43] Parhami-Seren B, Butenas S, Krudysz-Ambio J, Mann KG (2006). Immunologic quantitation of tissue factor. J Thromb Haemost.

[44] Key NS, Mackman N (2010). Tissue factor and its measurements in whole blood, plasma, and microparticles. Semin Thromb Hemost.

[45] Satta N, Toti F, Feugeas O, Bohbot A, Dachary-Prignet J, Eschwege V (1994). Monocyte vesiculation is is a possible mechanism for dissemination of membrane-associated procoagulant activities and adhesion molecules after stimulation by lipopolysaccaride. J Immunol.

[46] Dasgupta SK, Abdel-Monem H, Niravath P (2009). Lactadherin and clearance of platelet-derived microvesicles. Blood.

[47] Willekens FL, Werre JM, Kruijt JK, Le A, Bellera RV, Langlois K (2005). Liver Kupffer cells rapidly remove red blood cell-derived vesicles from the circulation by scavenger receptors. Blood.

[48] Al Faraj A, Gazeau F, Wilhelm C, Devue C, Guérin CL, Péchoux C (2012). Endothelial cell-derived microparticles loaded with iron oxide nanoparticles: feasibility of MR imaging monitoring in mice. Radiology.

[49] Rautou PE, Vion AC, Amabile N, Chironi G, Simon A, Tedgui A (2011). Microparticles, vascular function, and atherothrombosis. Circ Res.

[50] Thabut D, Tazi KA, Bonnefont-Rousselot D, Aller M, Farges O, Guimont MC (2007). High-density lipoprotein administration attenuates liver proinflammatory response, restores liver endothelial nitric oxide synthase activity, and lowers portal pressure in cirrhotic rats. Hepatology.

